# Factors That Impact Psychosocial Recovery 12 Months After Non-Severe Pediatric Burn in Western Australia

**DOI:** 10.3390/ebj7010005

**Published:** 2026-01-19

**Authors:** Amira Allahham, Dinithi Atapattu, Victoria Shoesmith, Fiona M. Wood, Lisa J. Martin

**Affiliations:** 1 School of Biomedical Sciences, University of Western Australia, 35 Stirling Highway, Crawley, WA 6009, Australiafiona.wood@health.wa.gov.au (F.M.W.); 2 Fiona Wood Foundation, 11 Robin Warren Drive, Murdoch, WA 6150, Australia; 3 Burn Service of Western Australia, Fiona Stanley Hospital, MNH (B) Main Hospital, Level 4, Burns Unit, 102-118 Murdoch Drive, Murdoch, WA 6150, Australia

**Keywords:** pediatric, burn, quality of life, psychosocial, outcome, family-centered care

## Abstract

**Background**: A childhood burn presents new and unfamiliar challenges to patients and their parents during recovery. These injuries can negatively impact activities such as independence in self-care, participation in physical activity, and social interaction. As such, pediatric burn patients are at risk of poorer quality of life (QoL) outcomes after their burn. In this longitudinal, observational cohort study, we examined the social, demographic, and clinical factors that were associated with a poor QoL at 12 months postburn for pediatric patients aged > 2 years with non-severe burns in Western Australia. **Methods**: Inpatients were recruited from the pediatric burn unit at Perth Children’s Hospital in Western Australia between February 2021 and September 2022. Demographic and family information (age, sex, postcode, parental education, languages spoken at home) and clinical data (burn cause, TBSA%, location, surgical interventions, length of stay) were collected at baseline. At 6 and 12 months, caregivers completed the Brisbane Burn Scar Impact Profile (BBSIP). **Results**: A total of 37 caregivers completed the Brisbane Burn Scar Impact Profile (BBSIP). For the child’s QoL, 57% of caregivers reported that some impact remained for overall QoL, 32% for sensory intensity, 46% for sensitivity, 22% for daily living (22%), and 19% for emotional reactions. Parent worry was impacted in 46% of caregivers. Being female was associated with greater long-term impacts, particularly in overall functioning and parental worry. The burn location also influenced outcomes, with injuries to the upper limbs linked to higher sensory intensity and emotional impact. Children from culturally and linguistically diverse (CaLD) backgrounds, indicated by those speaking a language other than English at home (LOTE), demonstrated significantly greater effects across several domains, including overall impact, daily living, appearance, and parent worry. **Conclusions**: A substantial proportion of children continued to experience impacts from non-severe burns across multiple domains, indicating that even small-area burns can have lasting effects. The factors associated with worse scores were the child being female, the families being linguistically diverse, and upper body burns.

## 1. Introduction

Childhood burns are stressful for patients and parents, especially mothers, in the acute stage of injury [1] and in long-term recovery [2], and this remains true for those with non-severe burns [3]. A 30-year study of more than 11,000 children admitted for burn injuries in Western Australia showed that patients are up to five times more likely to be admitted to hospitals for mental health conditions in the long-term following their burn admission, compared to the general population, despite the majority of patients having non-severe burns in this cohort [4]. This is supported widely in the literature, with pediatric burn patients and their parents reporting elevated levels of psychopathology and psychological symptoms, with reductions in quality of life both in the acute stages of the burn and in the long term [5,6,7].

Although psychosocial support and educational programs are often provided for burn patients and families, certain clinical, demographic, and social factors may put some patients at a higher risk of poor psychosocial and physical recovery than others. Clinically, greater burn severity (greater TBSA and burn depth) might negatively impact recovery [8], and there is a complex interplay between burn severity, outcomes, and demographic factors, such as age, sex, and other social factors [9]. For parents, if their child is female, they are reported to experience higher levels of post-traumatic stress disorder (PTSD) and distress, and parents of boys are found to have more symptoms of avoidance [10]. Additionally, there are indications that parents prioritize appearance over function for girls [11]. A minority race was shown to have a strong correlation with parent distress [12]. Other social factors, such as a lack of social support and financial burdens, have negatively impacted parental coping [13]. If a common profile of patients and parents who might be at risk of poor psychosocial outcomes can be outlined, then the families who would benefit from interventions to optimize psychosocial recovery can be identified early. This study aims to assess if factors identified at the initial presentation could explain poorer psychosocial outcomes at 12 months postburn.

## 2. Materials and Methods

This was a longitudinal, observational, cohort study that assessed the factors influencing psychosocial outcomes 12 months after a childhood burn. This analysis formed part of a larger prospective observational cohort study. Patients two years or older, and their parents or primary caregivers, who were admitted to the pediatric burn unit at Perth Children’s Hospital for the treatment of an acute non-severe burn (<20% TBSA) between 1 February 2021 and 30 September 2022 were invited to participate. Patients treated only in the outpatient clinic were not eligible to participate, as a proxy measure of burn severity. Children under 2 years were excluded because quality of life measures used in the parent study were not validated for this age group. The study size for this analysis was determined by the recruitment period and the attrition rate. Ethics approval was obtained from the Child and Adolescent Health Service Human Research Ethics Committee (HREC) at Perth Children’s Hospital (RGS 3310). The de-identified data presented in this study is available on request, and the data is not publicly available due to HREC privacy requirements. This study was conducted in accordance with the principles stated in the National Statement on Ethical Conduct in Human Research (NHMRC) and Good Clinical Practice (GCP) guidelines (ICH-GCP). This manuscript is reported in accordance with STROBE guidelines for observational studies.

### 2.1. Demographic, Clinical, and Social Factors

The demographic information gathered included the age at the time of injury (in years), sex (male or female), and residential postcodes. Parents self-reported family factors, such as languages other than English spoken at home, and some information about the circumstances of the injury. Clinical details were extracted from medical records and encompassed the burn location on the body, total body surface area affected (TBSA%), burn depth, burn cause (scald, flame, contact, friction, or other), and type of acute surgical intervention required (split-thickness skin graft, ReCell^TM^ (Avita Medical, Valencia, CA, USA) scaffold, other, or a combination).

### 2.2. Injury Event Questions

The following information was collected from parents at recruitment based on themes related to the impact on parents after a burn injury to their child [14]; the questions were originally adapted from a study by Winston et al. in 2003 [15]:

1. Did you see the incident (accident) in which your child got hurt?
2. When your child was hurt (or when you first heard it had happened) did you feel really helpless, like you wanted to make it stop but couldn’t?
3. Were you with your child in the ambulance or helicopter on the way to the hospital?
4. Does your child have any behavior problems or problems paying attention?

### 2.3. Quality of Life

Long-term outcomes were determined with the caregiver-reported Brisbane Burn Scar Impact Scale. This instrument measures nine separate domains of health-related quality of life outcomes for pediatric burn patients—overall impact, sensory intensity, mobility, daily living, daily routine, friendships and social interaction, appearance, emotional reactions, and physical symptoms—and two domains for parents or caregivers—parent worry and parent impact. There are two versions used in this analysis: The Caregiver Report for Children Aged Less Than 8 Years (58 items) and The Caregiver Report for Children Aged 8 Years and Older (61 items). These are validated, reproducible, and sensitive to change for most domains [16,17,18,19]. They take around 15 min to complete. These were collected at 6 and 12 m postburn. Caregiver-reported outcomes may be subject to social desirability and recall bias, with additional potential for selection bias and residual confounding.

### 2.4. Analysis

A descriptive analysis was completed to assess the proportions or percentages of categorical variables and medians and interquartile ranges for the continuous variables. Comparisons of paired medians were completed with the Wilcoxon signed-rank test to assess if there was a change overtime for the BBSIP. This was found to be stable between the 6- and 12-month timepoint, allowing the use of either timepoint as the long-term outcome. Thus the 12-month data was used preferentially, and the 6-month data was used for one patient when the 12-month data was missing.

The impact for each domain of the BBSIP at 12 months was dichotomized into ‘No impact’ (for those who select the ‘Not at all’ option) and ‘Some impact’ (for any other answer) and classified as long-term outcomes. This was performed to focus on the key question if there was any impact on quality of life and helped to stabilize the model for the small sample size. Logistic regression was used to assess the relationships between these domains and potential demographic, clinical, and social explanatory factors at baseline. Factors with a *p*-value < 0.2 were included in the initial model before a backward elimination. The backward elimination progressively drops variables if the *p* > 0.05 [20] to leave only the relevant adjusted explanatory variables in the model. The model fit was assessed using the area under the receiver operating characteristic curve (AUC) and McFadden’s Pseudo-R^2^. The AUC, which ranges from 0.5 (no discrimination) to 1.0 (perfect discrimination), indicated the model’s ability to distinguish between outcome categories, with values above 0.7 considered acceptable and values over 0.8 considered to be good. The Pseudo-R^2^, ranging from 0 (no improvement) to 1 (perfect fit), reflects the model improvement over the null model, with values above 0.2 viewed as indicative of a meaningful fit for logistic regression. The sensitivity to the stepwise inclusion threshold is implicitly addressed by the reporting of the univariate associations and final model retention. There were no subgroups analyzed. All analyses were completed in Stata16 [21]. The Stata code, full regression analyses, and forest plot illustrations are detailed in the Supplementary Information (File S1).

## 3. Results

### 3.1. Demographic and Injury Characteristics

This study recruited 51 patients overall; however, only 37 completed the long-term follow-up. Attrition was mostly due to injury recovery and discharge from clinical follow-up. The results focus on these patients. The demographic and injury characteristics are shown in Table 1. The sex, age, and TBSA of the patient were not different for those who did and did not complete this study up to the 12-month timepoint.

Languages other than English (LOTE) spoken in the home reflected a rich cultural diversity, highlighting the varied linguistic backgrounds of the participants. The majority were South Asian languages, including Gujarati, Hindi, Kannada, Konkani (with Hindi), Malayalam, and Tamil. Other languages included Cantonese (East Asia), Indonesian (Southeast Asia), and Polish (Europe).

For wound closure surgery, 3 patients (8%) received a split-thickness skin graft (STSG), 19 patients (51%) received an autologous skin cell spray graft (ReCell^TM^), and the remaining 11 patients (30%) had a combination of these. One patient had a negative pressure device over the STSG, and one patient had Biobrane^TM^ (Smith & Nephew, Watford, UK) applied over ReCell™.

For the questions about the injury event, 20 (54%) did not witness the incident. Most parents (31/37, 84%) reported feeling helpless at the time of the injury. Most children (32/37, 87%) were not perceived by parents to have behavioral or attention problems. Lastly, only eight (22%) of the parents accompanied their child to the hospital. 

### 3.2. Domain Analyses

The aim of this study was to assess what factors that were identifiable at the initial presentation for an acute burn injury could explain poor psychosocial outcomes at 12 months postburn. The overall long-term impact was measured with the BBSIP. For each domain, we describe the factors that met the criteria for inclusion into the multivariate analyses. The full analysis of all univariate analyses and stepwise multivariate analysis tables for each domain are found in the Supplementary Information Files (File S1).

#### 3.2.1. Overall Impact

Some impact was reported for the Overall Impact domain at 12 months for 57% (21/37) of patients. The initial model contained sex, metro residence, speaking a language other than English (LOTE) at home, and exposure to injury event question 3. After backward elimination, the multivariate analysis showed that, after adjusting for the child being female, speaking a language other than English was significantly associated with a higher long-term overall impact (OR = 11.2, 95% CI: 1.22–102.0, *p* = 0.033). For the nine families who spoke another language at home, eight reported some impact (89%). And for the 17 families whose child was female, 12 reported some impact (71%). The regression model fit is acceptable (AUC 0.75), and the model explains 17% of the variation in the overall impact (R^2^ = 0.1719).

#### 3.2.2. Sensory Intensity

Sensory intensity describes altered sensations in the scar, such as discomfort, tightness, itching, or pain. Some impact was reported for the Sensory Intensity domain at 12 months for 32% (n = 12) of patients. The initial model contained sex, languages other than English spoken in the home, and burns affecting the head or neck. After the backward elimination, the multivariate analysis showed that, after adjusting for the child being female, the sensory intensity was significantly higher if the family spoke another language in the home (OR = 10.5, 95% CI: 1.81–60.3, *p* = 0.009). For these nine families, six reported more sensory symptoms (67%). The regression model fit is acceptable (AUC 0.77), and the model explains 21% of the variation in the overall impact (R^2^ = 0.2171).

#### 3.2.3. Sensitivity

Sensitivity describes if the scar is sensitive to a light touch or clothing. Some impact was reported for this domain at 12 months for 46% (16/37) of patients. The initial model contained sex, age, and exposure to injury event question 2. After backward elimination, the multivariate analysis showed that none of these factors were significant to an α < 0.05. The model fit is acceptable (AUC 0.71), and the model explains 14% of the variation in the overall impact (R^2^ = 0.1377).

#### 3.2.4. Mobility

At 12 months, no parents reported a long-term impact on mobility for this cohort.

#### 3.2.5. Daily Living

This domain assesses impacts on activities such as schoolwork, play, dressing, bathing, eating, drinking, self-care activities, and general routine. Some impact was reported for the Daily Living domain at 12 months for 22% (8/37) of patients. The initial model contained languages other than English spoken in the home and burns affecting the chest, hand, or foot. After backward elimination, the multivariate analysis showed that, after adjusting for the location on the body, speaking another language in the home was associated with a significantly greater effect on long-term daily living (OR = 11.1, 95% CI: 1.06–115, *p* = 0.045). This was not influenced by being female. The regression model fit is good (AUC 0.85), and the model explains 35% of the variation in the overall impact (R^2^ = 0.3477).

#### 3.2.6. Friendships and Social Interaction

Only one child (2.7%) was reported by their parent to have difficulties with friendships and social interaction following the burn injury. All other children (36/37) were reported to have no difficulties in this area. The child reported to have difficulties was a 2-year-old male who sustained a 1.5% total body surface area (TBSA) scald to the arm and underwent surgical treatment. Due to the extremely low prevalence of reported difficulties in this domain, regression modeling was not appropriate. No inferential statistics are presented for this domain.

#### 3.2.7. Appearance

Three children (8%) were reported by their parent to have been bothered by their appearance (Table 2). Regression modeling was not appropriate for this event rate. All were scald injuries that required surgery to heal, and all children spoke a different language in the home.

#### 3.2.8. Emotional Reactions

This domain involves the parent’s opinion of the child’s emotional state about their burn; for example, it assesses irritability, anxiety, worry, sadness, depression, low confidence, anger, self-consciousness, and being upset. Some impact was reported for the Emotional Reaction domain at 12 months for 19% (7/37) of patients. The total predicted score, arm involvement, and TBSA were in the initial model. After backward elimination, the multivariate analysis showed that a unilateral arm burn was associated with a significantly greater effect on long-term daily routines (OR = 8.7, 95% CI: 1.3–53 *p* = 0.025). The regression model fit was acceptable (AUC 0.7190), and the model explains 15% of the variation in the overall impact (R^2^ = 0.1497).

#### 3.2.9. Parent Worry

This domain asks the parents if they are concerned about future impacts on the child, the effect on the family, and concerns about how others might treat their child. Some impact was reported for the parent worry domain at 12 months for 46% (17/37) of parents. The child being female and other languages spoken in the home were the two factors included in the initial model, and neither were removed with the backward elimination. The multivariate analysis showed that, after adjustment, speaking an LOTE was significantly associated with greater long-term patient worry (OR = 31.6, 95% CI: 2.8–361, *p* = 0.006), and the child being female was associated with greater patient worry (OR = 8.3, 95% CI: 1.38–50.1, *p* = 0.021). Of the 17 families where the patient was female, eleven parents (65%) reported feeling worried. Of the nine families where another language was spoken in the home, eight parents reported themselves to be worried (8/9). The regression model fit was good (AUC 0.83), and the model explains 32% of the variation in the overall impact (R^2^ = 0.3152).

#### 3.2.10. Parent Impact

This domain asks whether the scars impact the parent’s ability to work, study, and do household tasks; their relationship with the family; getting together with friends; their mood; and the family routine. Some impact was reported for the parent impact domain at 12 months for 19% (7/37) of parents. Injury event questions 1 and 4 and the involvement of the arms were included in the initial model, however none met the criteria (α < 0.05) for retention in the final multivariate model. Therefore, no specific demographic, clinical, or social factor appeared to influence the parent impact in this cohort.

## 4. Discussion

This study investigated the long-term impacts of burn injuries in children and sought to identify demographic and clinical factors at presentation that might be associated with poorer quality of life outcomes at 12 months post-injury. It is important to note that all burns in this study were non-severe, with none of them characterized by a burn area greater than 12% TBSA, and despite this the burn had an impact on the overall quality of life at 12 months postburn for more than half of the patients and their families. This study found that there were three potential factors that may explain poorer QoL outcomes after pediatric burns: the child being female, the burn location on the body, and the family speaking a different language at home. Interestingly, the event questions were not identified as a factor explaining quality of life at 12 months for these patients.

The child being female was significantly associated with increased parent worry in this study. Parental worry is assessed in the BBSIP using questions that focus on concerns about the scars bothering the child, the effect on other family members, and how others treated the child. This aligns with another local study that suggests that when parents were asked about their priorities for their child’s burn recovery, parents of girls prioritized appearance, and parents of boys prioritized function [11]. Mothers reported higher guilt scores when the patient was female [22,23], and parent distress was significantly associated with the child being female at the acute and mid-term follow-up [12]. Parents of female children were 3.6 times as likely to report a sub-optimal outcome for parental concern in a Dutch study [24]. Parental attitudes have been shown to differ towards children of different sexes as part of societal norms, where body image is seen to be more important for girls [25]. In addition, psychosocial outcomes reported by parents for their child can be biased due to heightened concern, post-traumatic stress, and feelings of guilt [22,26]. An earlier study by this group showed greater disparities between parent-reported and patient-reported psychosocial function scores of the pediatric quality of life questionnaire (PedsQL) for female patients at 3–9 months postburn [27]. This study supports these ideas: the parents of female burn patients worry more about their child compared to parents of boys; however, they did not report the current QoL status of the child as being different for male or female patients in other domains. Furthermore, the parents’ daily lives, mood, and relationships (parent impact domain) were impacted more broadly for one in five parents by their child’s scar, but this was not associated with the sex of the child. This suggests that parents’ worries might be more about their daughters’ futures rather than about their experiences of the current situation.

Research conclusions about how the effect of scarring differs by the sex of the child are mixed. An American study that compared the body esteem of burn-injured children with a non-burn control group found no difference between groups for boys, and better body esteem for burn-injured girls compared to the non-burn female controls [28]. A Dutch study reported that girls were 1.5 times more likely to perceive their (surgical) scars negatively and 4 times more likely to experience embarrassment about their scars compared to boys [29]. For adult patients, female sex and the importance of appearance were shown to be risk factors for developing body image dissatisfaction after burns [30]. Female burn patients are also more likely to experience longstanding impacts on their interpersonal and sexual relationships compared to males [31]. An Australian study showed that females burnt as a child were more likely to report long-term depression, anxiety, and any DSM-IV disorder compared to their male counterparts [32]. As such, the heightened parental worry in this study for pediatric female burn patients might be associated with societal attitudes towards the importance of body image, sexuality, and social and romantic relationships for their daughter’s future.

The involvement of the upper limb was associated with a significantly increased impact on emotional reactions (such as irritability, anxiety, sadness, and low confidence) (*p* = 0.045). The burn location is not a factor that has been widely explored in the literature, apart from exploring the impact of visible burn sites such as hands and faces [8,24,33]. However, it is important to note that this was independent of burn severity, as indicated by the TBSA. This is contrary to previous studies that indicated that QoL scores decrease with a greater TBSA and depth for both children and adults [24,34,35]. A Dutch/Flemish pediatric study found that children with burns with a TBSA over 10% had a significantly lower quality of life, particularly in relation to appearance, parental concern, and upper extremity function [24]. Another study linked a greater burn severity with increased maternal stress, which in turn increased the child’s stress [1]. It is important to note that non-severe burns also have a profound effect on QoL [36]. In this study, the limited range and low variability of the TBSA may have limited the statistical power, preventing the detection of a significant relationship.

Speaking a language other than English at home was an important factor associated with worse long-term QoL outcomes across several domains. In the Overall Impact domain, children from LOTE households were significantly more likely to experience greater long-term impacts, with eight of nine LOTE families (89%) reporting some level of impact. Although the sensory intensity domain did not retain LOTE as a significant factor, two-thirds of LOTE families (67%) reported altered sensations, such as discomfort, tightness, itching, or pain. In the daily living domain, which assesses any impact on activities such as schoolwork, play, bathing, and self-care, speaking another language in the home was significantly associated with greater long-term impacts on daily living. Within the appearance domain, all three children reported as being bothered by their appearance came from LOTE households. Similarly, for parent worry, speaking a language other than English at home was strongly associated with greater long-term parental concern, with eight of nine parents (89%) reporting ongoing worry. In the overall impact and parent worry domains, being female and speaking a language other than English in the home were independently associated with poorer outcomes. This pattern may indicate an additive or interacting influence, whereby sociocultural and gender-related factors jointly contribute to increased vulnerability following a burn injury. Unfortunately, the sample size in this study was insufficient to allow the inclusion of interaction terms in the multivariate models, and thus, these relationships should be interpreted with caution and explored further in larger, more diverse studies with enough statistical power to test interaction effects. It is important to note that this study recruited patients and parents who had a good understanding of the English language, as this was required for both informed consent and study participation. Given this, it is unlikely that participants had misunderstandings regarding the study material. As such, we suggest that the root cause of the significant impact observed in this group is likely associated with patients’ cultural backgrounds rather than speaking a different language at home, and the LOTE factor can be a proxy measure of differing sociocultural backgrounds.

There is limited literature investigating the impact of cultural and language differences on the outcomes of burn patients, especially in Australia. This might be due to the majority of studies in the Australian context being conducted mostly in English, with little options for interpreter involvement or translation. An Australian study found that pediatric burn patients who spoke a language other than English at home were more likely to need grafting for their burns [37]. Another study from the US found that patients with limited English proficiency were more likely to have an increased number of scheduled in-person follow-ups, which was suggested to be due to language barriers preventing phone follow-ups. In addition, these patients were more likely to return to the emergency department after discharge. This might indicate communication gaps leading to misunderstanding care plans or the reluctance by patients to seek phone advice due to language barriers [38]. Individuals from diverse linguistic backgrounds often face many barriers within the healthcare system, including the poor cultural competency of healthcare providers, structural disadvantages and vulnerabilities, the inadequate inclusion of migrants and refugees’ health in regional organizations, and poor health system responses for people from such populations [39]. Such barriers may increase the psychosocial impact on burn patients and their families.

The experience of pain and sensations is known to vary across race and sex [40]; therefore, cultural backgrounds and sex may have an influence on perceived sensitivity or on the factors that contribute to sensitivity. The daily living factor describes the impact of the burn on activities such as dressing, eating, bathing, playing, and school [16]. These activities vary across cultures and social contexts. One study found that socioeconomic factors, including a family’s cultural emphasis, household activities, the occupational prestige of the household head, and larger family sizes, affected social competence after burns [29]. This suggests that household and cultural dynamics influence a child’s reintegration into social and school activities. Another qualitative study reported that while children were happy to return to school, they often felt excluded or embarrassed about their burns and pressure garments and frustrated by difficulties managing personal care tasks such as bathing and dressing [30]. A review of burn outcomes in the US found that racial minorities generally experienced poorer inpatient and outpatient outcomes. However, language was not examined as an independent factor, despite being a common characteristic across many racial groups studied (e.g., Asian, Hispanic, Latino, Indigenous) [41]. In addition, parental post-traumatic stress symptoms were associated with a reduced QoL of the patients and a racial minority status, where parents from racial minorities had higher levels of post-traumatic stress symptoms than parents from non-minority races [3].

In summary, at 12 months post-injury, a substantial proportion of children continued to experience impacts from non-severe burns across multiple domains, indicating that even small-area burns can have lasting effects. Being female was consistently associated with greater long-term impacts, particularly for overall functioning and parental worry. The burn location also influenced outcomes, with injuries to the upper limbs linked to higher sensory intensity and emotional impact. Children from culturally and linguistically diverse (CaLD) backgrounds, indicated by those speaking a language other than English at home, demonstrated significantly greater effects across several domains, including the overall impact, daily living, appearance, and parent worry. Together, these findings highlight that the psychosocial and functional recovery following pediatric non-severe burns is shaped not only by clinical factors but also by the sex of the child and the cultural context.

The small sample size limits statistical power. Small sample studies can inflate odds ratios, so the direction and significance are important rather than the magnitude. The large odds ratios and wide confidence intervals suggest imprecision due to the small sample size; however, they remain statistically significant. Some variables and interaction terms could not be reliably estimated due perfect predictions. The generalizability of these findings is limited by the small, single-site design, and the results may not be representative of broader populations or other settings.

## 5. Conclusions

Pediatric burn patients are particularly vulnerable to poor psychosocial outcomes due to the stress and the personal limitations that accompany the recovery process from burn injuries. In this study, we have investigated the clinical, demographic, and social factors influencing psychosocial outcomes of pediatric burn patients 12 months after their injuries through quality of life questionnaires and questions about the injury and recovery, which were all completed by the patients’ parents. Our findings showed that being female and speaking a language other than English at home were the two main predictors of poor psychosocial outcomes after non-severe pediatric burn injuries, especially for domains such as parental stress, mobility, and daily routines. We suggest that these factors are associated with the higher anxiety of the parents of pediatric burn patients who are female or who come from diverse cultural backgrounds. Factors such as worries about appearance and limited understanding in a health setting could also contribute. Hence, we recommend that there should be a lower threshold for offering psychosocial interventions and support services to pediatric burn patients and their families when the child is female and/or from culturally diverse backgrounds.

## Figures and Tables

**Table 1:** Patient characteristics.

Sex	Male	n (%)	20 (54%)
Age	Years	median (IQR) range	6 (7) 2–15
Area of residence	Metro	n (%)	30 (81%)
TBSA	% burn	median (IQR) range	2.5 (3.4) 0.2–12
Burn type	Scald	n (%)	24 (65%)
	Contact	n (%)	8 (22%)
	Flame	n (%)	3 (8%)
	Friction	n (%)	1 (3%)
	Electrical	n (%)	1 (3%)
Surgery for wound closure		n (%)	33 (89%)
Location of burn on body	Head/neck	n (%)	6 (16%)
	Trunk	n (%)	16 (43%)
	Arm(s)	n (%)	9 (24%)
	Hand(s)	n (%)	5 (14%)
	Leg(s)	n (%)	19 (51%)
	Foot/feet	n (%)	10 (27%)
LOTE		n (%)	9 (24%)

**Table 2:** Characteristics of patients whose parents report some impact from the child being bothered by their appearance.

Age	Sex	Burn Type	Location	TBSA	Surgery	LOTE
2	Female	Scald	Head/neck, chest	4.5	Yes	Yes
5	Female	Scald	Chest, right arm	3	Yes	Yes
7	Male	Scald	Bilateral legs	5	Yes	Yes

## Data Availability

The data are not publicly available due to HREC privacy requirements. The de-identified data presented in this study are available on request from the corresponding author. The applicant requires the appropriate ethics permissions.

## Supplementary Materials

The following supporting information can be downloaded at https://www.mdpi.com/article/10.3390/ebj7010005/s1: File S1: Supplementary Information contains Stata Code, Univariate Analyses and Stepwise Multivariate Analysis Tables for each domain.
