# Supplementary material for: Factors That Impact Psychosocial Recovery 12 Months After Non-Severe Pediatric Burn in Western Australia
Source: Eur Burn J. 2026 Jan 19;7(1):5. doi: 10.3390/ebj7010005 (PMC12922029; doi:10.3390/ebj7010005)
Supplement: Supplementary file 1 [file ebj-07-00005-s001.zip › ebj-4044149-supplementary/ebj-4044149-supplementary.pdf]

File S1: Supplementary Information contains Stata Code, Univariate Analyses and Stepwise Multivariate Analysis Tables for each domain

Stata code format:

```
logit LTBBSIP# `var', vce(robust) nolog or
stepwise, pr(0.05) pe(0.04) lockterm1: LTBBSIP# (0.sex) varlist(sigvar), vce(robust)
nolog or
stepwise, pr(0.05) pe(0.04: LTBBSIP# varlist(sigvar), vce(robust) nolog or
```

where: # = domain number; `var' = variable; varlist = variable list; sigvar=variables identified in univariate analysis for inclusion in full multivariate model. Note: Univariate analyses show ORs for 'male'. In the multivariate analyses these tables display the ORs for 'female'. This is a binary variable that records 'sex of the child at birth'. Thus, these are equivalent.

Domain 1 – Overall Impact

Table 1 Univariate Analyses (overall impact domain)

Variable	OR	Robust SE	95% LCL	95% UCL	p-value
Male	0.34	0.24	0.085	1.361	0.128
Metro Residence	4.32	4.02	0.696	26.78	0.116
LOTE	9.23	10.5	0.985	98.53	0.052
Predictor 3	0.15	0.18	0.015	1.553	0.112

Table 2 Stepwise Multivariate Analysis (overall impact)

Variable	OR	Robust SE	95% LCL	95% UCL	p-value
LOTE	9.2	10.5	0.985	98.5	0.052

Domain 2 – Sensory Symptoms

Table 3 Univariate Analyses (sensory symptoms domain)

Variable	OR	Robust SE	95% LCL	95% UCL	p-value
Male	0.28	0.211	0.065	1.225	0.091
Head/neck burn	5.75	5.587	0.856	38.6	0.072
LOTE	7.33	6.274	1.371	39.22	0.020
Burn type	Overall test: chi2(1) 5.42				0.019

Table 4 Stepwise Multivariate Analysis adjusting for sex (sensory symptoms domain)

Variable	OR	Robust SE	95% LCL	95% UCL	p-value
LOTE	16.8	20.43	1.554	181.9	0.020
Female	4.79	5.80	0.446	51.42	0.196

Table 5 Stepwise Multivariate Analysis not adjusting for sex (sensory symptoms domain)

Variable	OR	Robust SE	95% LCL	95% UCL	p-value
LOTE	9.00	9.416	1.158	69.955	0.036

Domain 3 – Sensitivity

Table 6 Univariate Analyses (sensitivity domain)

Variable	OR	Robust SE	95% LCL	95% UCL	p-value
Male	0.28	0.201	0.067	1.146	0.076
Q2	5.36	6.287	0.537	53.45	0.153
age	1.118	0.097	0.943	1.325	0.199

Table 7 Stepwise Multivariate Analysis (sensitivity domain)

Variable	OR	Robust SE	95% LCL	95% UCL	p-value
-	-	-	-	-	-

Domain 4 – Mobility

No impact reported for this domain.

Domain 5/6 – Daily Living/daily routine

Table 8 Univariate Analyses (daily living domain)

Variable	OR	Robust SE	95% LCL	95% UCL	p-value
LOTE	4.80	4.191	0.867	26.57	0.072
chest	14.4	13.93	2.181	95.65	0.006
hand	8.10	9.496	1.037	63.28	0.046
foot	7.67	7.287	1.190	49.39	0.032

Table 9 Stepwise Multivariate Analysis (daily living domain)

Variable	OR	Robust SE	95% LCL	95% UCL	p-value
LOTE	11.06	13.3	1.057	115.6	0.045
chest	27.6	33.2	2.599	292.3	0.006

Domain 7 – Friendship and social interaction

One child was reported by their parent to have difficulties with friendships and social interaction following the burn injury.

Domain 8 – Appearance

Three children were reported by their parent to have been bothered by their appearance, and therefore regression modelling was not appropriate.

Domain 9 – Emotional reactions

Table 10 Univariate Analyses (emotional reactions domain)

Variable	OR	Robust SE	95% LCL	95% UCL	p-value
TBSA	1.26	0.176	0.963	1.659	0.092
arm	8.3	7.89	1.300	53.40	0.025
Q1	0.15	0.169	0.015	1.406	0.096
Predict score	0.41	0.191	0.166	1.024	0.056

Table 11 Stepwise Multivariate Analysis (emotional reactions domain)

Variable	OR	Robust SE	95% LCL	95% UCL	p-value
arm	8.33	7.898	1.300	53.40	0.025

Domain 10 – Parent Worry

Table 12 Univariate Analyses (parent worry domain)

Variable	OR	Robust SE	95% LCL	95% UCL	p-value
Male	0.23	0.167	0.058	0.947	0.042
LOTE	16.89	19.44	1.769	161.1	0.014

Table 13 Stepwise Final Multivariate Analysis (parent worry domain)

Variable	OR	Robust SE	95% LCL	95% UCL	p-value
LOTE	31.6	39.25	2.758	361.2	0.006
Female	8.32	7.62	1.38	50.11	0.021

Domain 11 – Parent Impact

Table 14 Univariate Analyses (parental impact domain)

Variable	OR	Robust SE	95% LCL	95% UCL	p-value
arm	3.60	3.316	0.592	21.89	0.164
Q1	0.15	0.169	0.015	1.406	0.096
Q4	3.75	3.449	0.618	22.75	0.151

Table 15 Stepwise Multivariate Analysis (parent impact domain)

Variable	OR	Robust SE	95% LCL	95% UCL	p-value
-	-	-	-	-	-
